# Hierarchical Inference in Sound Change: Words, Sounds, and Frequency of Use

**DOI:** 10.3389/fpsyg.2021.652664

**Published:** 2021-08-12

**Authors:** Vsevolod Kapatsinski

**Affiliations:** Department of Linguistics, University of Oregon, Eugene, OR, United States

**Keywords:** hierarchical inference, sound change, lexical diffusion, frequency effects, usage-based phonology

## Abstract

This paper aims examines the role of hierarchical inference in sound change. Through hierarchical inference, a language learner can distribute credit for a pronunciation between the intended phone and the larger units in which it is embedded, such as triphones, morphemes, words and larger syntactic constructions and collocations. In this way, hierarchical inference resolves the longstanding debate about the unit of sound change: it is not necessary for change to affect only sounds, or only words. Instead, both can be assigned their proper amount of credit for a particular pronunciation of a phone. Hierarchical inference is shown to generate novel predictions for the emergence of stable variation. Under standard assumptions about linguistic generalization, it also generates a counterintuitive prediction of a U-shaped frequency effect in an advanced articulatorily-motivated sound change. Once the change has progressed far enough for the phone to become associated with the reduced pronunciation, novel words will be more reduced than existing words that, for any reason, have become associated with the unreduced variant. Avoiding this prediction requires learners to not consider novel words to be representative of the experienced lexicon. Instead, learners should generalize to novel words from other words that are likely to exhibit similar behavior: rare words, and the words that occur in similar contexts. Directions for future work are outlined.

## Introduction

Research on sound change has been characterized by a tension between the fact that changes affect specific sounds in phonological contexts, and the fact that changes progress faster in some words and expressions than in others. For example, a final post-consonantal /t/ is likely to be deleted in American English, compared to other comparable sounds like /k/ or /p/. At the same time, this deletion is more likely in a frequent word like *most* than in an infrequent word like *mast* (Bybee, 2002). These facts appear to be in conflict because approaches to sound change tend to assume that there is a particular unit of change, which is either the sound – in approaches growing out of the Neogrammarian tradition (Osthoff and Brugmann, 1878; Labov, 1981) – or the word, in the dialectological / lexical diffusion tradition where every word has its own history (Schuchardt, 1885; Mowrey and Pagliuca, 1995).

For example, generative grammatical theory (Chomsky and Halle, 1965), and allied approaches in psycholinguistics (Levelt, 1989; Levelt et al., 1999) have suggested that the long-term representations of words are composed of a small set of discrete segments (whether phones, features or syllables). In this architecture, words are not directly associated with specific pronunciations, and therefore the pronunciation of a segment is not lexically specific. As a result, only two types of sound change are possible – a phonetically abrupt deletion, insertion or substitution of a segment in the lexical representation of a particular word, or a continuous drift in the pronunciation of a particular segment that happens across all instances of the segment in a particular phonological environment, no matter what word it is embedded in Labov (1981). This theory has difficulty explaining how words can influence the pronunciation of a segment in a gradient manner (Bybee, 2002). For example, the durations of frequent words are shorter than the durations of homophonous infrequent words (Gahl, 2008). On the opposite end of the spectrum is Mowrey and Pagliuca’s (1995) proposal that words are holistic motor programs specifying the timing and intensity of nerve impulses to muscles controlling articulator movement. This approach allows for each word to have its own history, and for lexical representations to change continuously rather than in discrete jumps (Mowrey and Pagliuca, 1995; Bybee, 2001, 2002). However, it has the converse problem of being unable to explain why a word’s pronunciation does not change uniformly, i.e., why certain sounds are affected more than others.

Pierrehumbert (2002) unifies the segmental and lexical views of sound change by suggesting that the language system maintains representations of segmental categories, which are implemented as sets of exemplars, but that each exemplar of a segment is tagged with the word in which it occurred. In production, the selection of a segment exemplar is then driven both by the identity of the segment and the identity of the word: both are tags available to cue an exemplar in production. A related idea is the approach to reduction proposed by Browman and Goldstein (1989) within Articulatory Phonology, where gestures are units of change but the timing and magnitude of a gesture can be lexically specific.

The present paper combines this idea with rational probabilistic inference (Xu and Tenenbaum, 2007; Feldman et al., 2009; Perfors et al., 2011; Kleinschmidt and Jaeger, 2015; O’Donnell, 2015; Harmon et al., 2021). If both the identity of a segment and the identity of the word that contains it influence the pronunciation of a segment in a lexical context, then a rational language learner would use hierarchical inference to allocate credit for a particular pronunciation between the two influencers. This paper explores the consequences of this assumption for articulatorily-motivated sound change.

I focus on articulatorily-motivated changes because the role of inference in such changes has been underexplored. In the other major type of change, analogical change, a role for inference is relatively uncontroversial (e.g., Bybee, 2001). In analogical changes, words (or other stored forms) that exemplify a minority grammatical pattern succumb to analogical pressure from the rest of the lexicon. Low-frequency words succumb to this pressure more readily than high-frequency words (Phillips, 1984, 2001; Lieberman et al., 2007; Todd et al., 2019). This is exactly what is to be expected from hierarchical inference. Because the learner has little evidence for the behavior of a rare word being idiosyncratic, such a word is likely to be mistakenly inferred to behave like a typical word (of the same type).

In contrast to analogical changes, articulatorily-motivated changes start in frequent words (Schuchardt, 1885; Fidelholtz, 1975; Hooper, 1976; Phillips, 1984, 2001; Mowrey and Pagliuca, 1987, 1995; Bybee, 2001). These are words with which the speaker has had the most practice. A change that targets a frequent word or phrase (like *going to* reducing to a nasal schwa in some contexts) cannot be due to the learner receiving insufficient evidence for the original, conservative pronunciation. Instead, these changes appear to be due to streamlining of articulation of a word or phrase with extensive practice. This conclusion is supported by the reductive character of such changes, which invariably involve temporal and/or substantive reduction of articulations, or smoothing out of the transitions between articulatory targets (Mowrey and Pagliuca, 1987, 1995; Browman and Goldstein, 1989; Bybee, 2001; Kapatsinski et al., 2020).

Most research on articulatorily-motivated sound change has not considered inference to play a role in this process. This would be appropriate if the progression of articulatorily-motivated changes were entirely mechanical, rather than partly governed by the conventions of the speech community. That is, if you could perfectly predict the degree of reduction in a context from the phonetics of the context – the articulatory routine being automatized – and the amount of practice that speakers had with it.

However, it is clear that this is not a tenable assumption. For example, Coetzee and Pater (2011) show that the rate of reducing /t/ or /d/ at the ends of words like *most* is affected by the following phonological context in different ways across varieties of English. This means that the rate of t/d reduction in a particular context needs to be learned as part of acquiring a particular variety of English. Certain segments are more likely to be reduced than others in a particular language with probability of reduction varying between languages (e.g., /k/ is reduced in Indonesian, /t/ in English, and /s/ in Spanish; Cohen Priva, 2017). Furthermore, the same segment in the same context can be reduced in different ways in different language varieties. For example, where Americans flap, many Brits would produce a glottal stop. Thus, a speaker needs to learn what to reduce, how to reduce, and when / in what contexts to reduce in part from exposure to what is done in their community.

As discussed above, articulatorily-motivated reductions are often particularly advanced in specific segments or gestures. For example, [t] is often reduced to the point of being deleted in *massed*, *mast* or *most* (Bybee, 2002; Coetzee and Pater, 2011) but [k] in *mask* or *musk* is not equally reduced. At the same time, such changes are also affected by the identities of the words in which the segment is embedded. Furthermore, some of this lexical conditioning is idiosyncratic, rather than attributable to word frequency, suggesting that the effects of word identity on pronunciation choices also need to be learned from exposure to the ambient language variety (Pierrehumbert, 2002; Wolf, 2011). For example, Zuraw (2016) mentions that the verb *to text* shows a particularly high rate of final [t] deletion. Because both segments and words affect pronunciation choices, a rational learner would use hierarchical inference to infer how much responsibility for a particular pronunciation rests at the lexical level.

### Contribution of This Paper

In this paper, I consider how automatization of articulation interacts with learning processes by which the listener infers when and what to reduce. The principal innovation of the present paper, in the context of the literature on sound change, is to model this learning process. In the proposed model, learning is understood as rational probabilistic inference. That is, the listener infers the likely combination of causes that resulted in a particular observed pronunciation. Crucially, this inference process is argued to be hierarchical in nature (Xu and Tenenbaum, 2007; Feldman et al., 2009; Perfors et al., 2011; Kleinschmidt and Jaeger, 2015; O’Donnell, 2015; Harmon et al., 2021).

As noted above, since the 1870s, research on sound change has been dominated by a debate between the Neogrammarian doctrine of regular sound change, in which the change affects all instances of a phonological structure at once (“sounds change”; Osthoff and Brugmann, 1878) and the doctrine of lexical diffusion, in which words change one by one, so that a sound change diffuses gradually through the lexicon (“words change”; Schuchardt, 1885). Hierarchical inference allows the proposed model to capture the insight that the answer is *both*. That is, the likelihood of producing a particular phone in a particular context is determined *both* by the phoneme it instantiates, and by the larger units in which it is embedded (Pierrehumbert, 2002). For example, even though a /t/, in the right phonological context, is generally very likely to be realized as a flap in American English, this likelihood is somewhat lower when the /t/ is embedded in the formal word *emitter*.

The model described here captures this effect of lexical identity on the choice of an articulatory target for a sublexical unit. It is intended as the simplest possible model incorporating hierarchical inference into a theory of sound change. The model is easily extendable to incorporate additional levels in the linguistic hierarchy as influences on pronunciation, such as phonological units above the segment, morphemes, or collocations, all of which influence pronunciation (Mowrey and Pagliuca, 1995). Speakers and speaker groups can also be incorporated as an additional random effect specifying knowledge of sociolinguistic variation to account for speakers’ ability to produce or imitate more than one dialect (e.g., Vaughn and Kendall, 2019).

A classic problem in sound change is why it does not always happen, even though the seeds for it are ever present (termed the *actuation problem* by Weinreich et al., 1968). Inference appears to play a crucial role in actuation. For a sound change to take off, an innovative pronunciation needs to be reproduced, both by the same speaker and by the speakers s/he talks to. Inference of the causes of the pronunciation appears to play an important role in this process. Specifically, experimental research has demonstrated unconscious imitation of phonetic detail, which shows how innovative productions can influence both future productions by the same speaker and those of their interlocutors (Goldinger, 1998). However, the extent and even direction of this influence can be affected by the listener’s perception of the reason for which the speaker produced the word in a novel way, or in an unfamiliar context. For example, when the speaker is perceived to not be a fully competent speaker of the language, or to be a carrier of a stigmatized dialect, the listener is less likely to imitate the production (Babel, 2012; see also Bannard et al., 2013; Oláh and Király, 2019). The speaker is also less likely to reuse a pronunciation that has received a negative evaluation by an interlocutor (Buz et al., 2016). The listener’s evaluation of a production, and therefore the spread of a change that originates in production, is thus influenced by a process of inference that identifies the production’s cause.

The aspect of actuation I focus on here is diffusion of an innovative pronunciation through the lexicon, rather than through the community of speakers. In this context, it is important for a listener who considers adopting a speaker’s pronunciation to know how far to generalize from the experienced examples. For example, observing *butter* produced with a flap, the listener might think that this is the way that the speaker pronounced *butter*, the way they pronounce the phoneme /t/, the way they pronounce an intervocalic /t/, etc. Depending on the structure(s) to which credit for the new pronunciation is assigned, a listener who decides to adopt the speaker’s innovation might confine it to the particular word in which it was observed, or generalize it to a larger subset of the vocabulary (see Xu and Tenenbaum, 2007, for the equivalent problem in generalizing a wordform to a specific Dalmatian, all Dalmatians or all dogs). Nielsen (2011) has shown that unconscious imitation generalizes beyond the experienced word to other instances of the same phone, and even other phones sharing phonological features with it. In order to know how far to generalize a pronunciation, the listener needs to infer what caused the speaker to produce it. It appears that not only do listeners make inferences about why a speaker pronounced a certain segment in a certain way (see also Marslen-Wilson et al., 1995; Kraljic et al., 2008), this inference also influences their likelihood of reproducing the pronunciation.

I show that hierarchical inference provides a novel perspective on the puzzling phenomenon of stable variation. Sometimes, the diffusion of an innovative pronunciation variant through the lexicon stalls, resulting in stable lexically specific variation. A classic example is *-ing* vs. *-in’* in English, which has been stable for decades. (Labov, 1989; Abramowicz, 2007; Gardiner and Nagy, 2017). Stable variation presents a challenge to exemplar-theoretic models of sound change (e.g., Pierrehumbert, 2001) because a consistent leniting bias should eliminate the conservative variant (Abramowicz, 2007). The proposed model accounts for how variation can remain stable, even if one of the variants is already statistically dominant, and articulatory pressures always favor the dominant variant. The proposed model is unique in making clear predictions about the conditions under which stable variation is likely to emerge, and the level at which variation is likely to stabilize (Sections “Inference of a Random Effect of Lexical Identity: Lexicalization, Polarization, Stable Variation and a U-Shaped Frequency Effect” – “Stable Variation Depends on the Frequency Distribution and Its Effect on Reduction”).

An important question begged by suggesting that the language learner takes words to be samples from a classified lexicon is whether the learner expect words s/he encounters in the future to be like the words she has already encountered? Or does s/he think that the words s/he is about to encounter might differ systematically from words s/he already knows (see Navarro et al., 2013, for the latter in learning non-linguistic categories)? In particular, if frequent words systematically differ from rare words, does the learner catch onto this fact, extrapolating that newly encountered (and therefore presumably rare) words are likely not to be like the frequent words s/he already knows (see also Baayen, 1993; Barth and Kapatsinski, 2018; Pierrehumbert and Granell, 2018)? This hypothesis is compatible with the widely adopted assumption that the grammar is primarily for dealing with novel inputs, with known words largely retrieved from memory (e.g., Bybee, 2001; Albright and Hayes, 2003; Kapatsinski, 2010a,b, 2018a). If the grammar is there primarily to deal with novel inputs, then it would be rational for the learner to base their knowledge of how to deal with novel inputs on experience with rare/novel inputs. Alternatively, learners may simply learn how known words and phones are pronounced without inferring anything about the relationship between word frequency and pronunciation. I take this to be the standard assumption in usage-based linguistics (e.g., Bybee, 2001: 12). The proposed model allocates the most likely amount of credit for a pronunciation to each of its *conceivable* causes, where causes are conceivable if they are considered by the listener. From this perspective, the question raised in the preceding paragraph reduces to whether conceivable causes of reduction likely include frequency of use. I will show that this is necessary for a monotonic relationship between frequency and reduction to be maintained after the reduced variant becomes dominant in the lexicon (Section “If Novel Words Are Thought to be Like Rare Words, Frequency Effect Will Stay Monotonic”).

### Relations to Other Work

The proposed model views language acquisition as a combination of automatization of production and rational probabilistic inference. Automatization is often discussed in work on sound change (Mowrey and Pagliuca, 1995; Pierrehumbert, 2001) as well as on the effects of experience on production (Tomaschek et al., 2018). Probabilistic inference is extensively explored in work on acquiring language from perceptual input (Xu and Tenenbaum, 2007; Feldman et al., 2009; Perfors et al., 2011; Kleinschmidt and Jaeger, 2015; O’Donnell, 2015). However, the interaction of the two mechanisms and its implications for the structure of language have remained unexplored.

Hierarchical inference conceptualizes sublexical units as classes of words sharing a particular chunk, and words are conceptualized as classes of utterances. This view of the nature of hierarchies aligns with the usage-based view of linguistic representations in considering linguistic units to be categories of experienced utterances (Bybee, 1985, 2001; Edwards et al., 2004), rather than building blocks out of which larger units are composed. For example, there is nothing in the proposed model that demands that an utterance be exhaustively parsed into morphemes. Whatever morphemes affect pronunciation choices are simply attributes shared by a class of words. Words sharing the morpheme *-ado* in Spanish are a class in the same way that Latinate words are a class in English. Even though the former are all similar in the same way, and the latter share no more than a family resemblance, both can affect pronunciation choices (e.g., lenition of [d] and stress placement, respectively). Despite the ‘hierarchical’ in the name, hierarchical inference does not require classes to form a strict hierarchy. In fact, hierarchical inference is compatible with any model of linguistic categorization that results in associable categories that share members. For example, the structural descriptions of rules in Albright and Hayes (2003) can also be considered word classes and are potentially subject to hierarchical inference. In Albright and Hayes (2003), rules associated with the same change can be nested, so that a more specific rule like “0→ed after a voiceless fricative” can co-exist with a more general rule like “0→ed after any consonant”. It is therefore possible for a learner to use hierarchical inference to allocate credit for a particular instance of *-ed* surfacing after a voiceless fricative across rules that enact the same change (see O’Donnell, 2015, for a model that does this in morphosyntax). However, the closest work to the present proposal in the literature is Pierrehumbert’s (2002) hybrid exemplar/generative model of sound change.

Pierrehumbert (2002) proposed that the speaker stores tokens of phones, and tags them with the identities of the words in which they occurred (as well as other contextual characteristics). From the present perspective, these tags define partially overlapping classes of pronunciation exemplars. Again, a strict hierarchy is unnecessary: the class of segment exemplars tagged with the word *cattish* and the class of exemplars tagged with /t/ can co-exist in the model even though not all /t/ exemplars occur in *cat* and even though exemplars tagged with *cattish* also include exemplars of other sounds. Selection of a pronunciation variant in producing a word is then biased to some extent by the identity of the word. The model proposed here builds on Pierrehumbert’s model by incorporating an inference mechanism, which infers the contribution of a particular class/tag to a particular pronunciation of a segment. This inference determines how much the tag should influence the pronunciation of the segment in the speaker’s subsequent production. In addition, by treating word identity and phonological context as independent influences on variant choice, the proposed model can account for cases in which reduced variants surface in phonological contexts that otherwise disfavor them. For example, Shport et al. (2018) show that American English speakers flap the /t/ in *whatever* even though flapping is otherwise illegal inside a word before a stressed vowel. By treating words as a random effect, the proposed model predicts such cases to be fairly rare and restricted to frequent words that are likely to be reduced and can resist the pull of the rest of the lexicon to regress to the mean but, crucially, does not predict them to be impossible. In addition, the present model generates stable variation and makes predictions about when it is likely to emerge.

## The Model

The most basic version of the model thus consists of the following parts:

(1) there are two pronunciation variants, reduced and unreduced;
(2) every time a word is used, the likelihood of the reduced variant of the phone being used in that word is incremented; as a result, reduction advances further in frequent words than in rare ones; and
(3) when a learner is exposed to the language, s/he learns not only an overall probability for each variant but also how variant probabilities are affected by lexical context.

In other words, the model proposes that the child learns how often a certain phone is pronounced a certain way and that some words are pronounced exceptionally. This kind of word-specific phonetic learning appears to be necessary because lexical frequency does not account for all between-word variability in phone pronunciation; a residue of exceptionality remains after frequency is accounted for Pierrehumbert (2002); Wolf (2011); Zuraw (2016).

The model assumes that the inference process is functionally equivalent to hierarchical regression. Below, it is implemented specifically as a logistic regression because of the first assumption above, the existence of alternative production targets associated with a phoneme in context such as an intervocalic /t/, which can be realized as a flap or a stop in American English. However, most reductive processes can also be conceived of as phonetically gradient rather than categorical (e.g., De Jong, 1998, for flapping; Bybee, 2002, for t/d deletion). Fortunately, the same predictions would be made by the present model if reduction were assumed to be continuous. We would simply replace the logistic link function with the identity link function of linear regression. Nothing hinges on the choice of the logistic linking function below.

The model was implemented in R (R Core Team, 2020) and is available at https://osf.io/qt6x4/. For ecological validity, I elected to simulate real sublexica that might be affected by a sound change. I considered two sublexica that are on the opposite ends of a productivity continuum: a large sublexicon with many rare words and a low maximum token frequency, and a small sublexicon with few rare words and a high maximum token frequency. The first sublexicon is the set of words with an intervocalic /t/ or /d/, followed by an unstressed vowel. The second sublexicon is the set of words beginning with eth (/ð/). Words in the first set constitute words in which the /t/ or /d/ is eligible to be flapped regardless of the broader context in American English (e.g., Herd et al., 2010). Words in the second set are eligible to undergo stopping in some dialects (e.g., Drummond, 2018), though this is not the full set of words eligible for stopping. However, our aim here is not to model these specific changes, but rather to ensure that the results of modeling are robust across sublexica that are maximally distinct in type frequency and the token/type ratio, which are the only characteristics of words that the model can see. Where noted, these sublexica are modified by excluding the most frequent words, those with frequency above 300, to explore the influence of these lexical leaders of change on its progression.

The first generation was seeded with one of two sublexica. The first sublexicon was the full sample of words eligible for flapping from the Switchboard Corpus (Godfrey et al., 1992). All words with a flapping context in the CMU Pronouncing Dictionary (Weide, 1995) were included (*N* = 762). These words had a stressed vowel followed by a /t/ or /d/ followed by an unstressed vowel. Each word occurred in the input with the frequency with which it occurred in the corpus, which followed the highly skewed Zipfian distribution (Zipf, 1935): 236 words were hapax legomena, occurring in the input only once; the most frequent word, *little*, occurred 2793 times.

The second sublexicon is the set of English words that start with /ð/. This set has far lower type frequency (only 24 distinct wordforms are found in Switchboard). It is also not Zipfian-distributed because it includes several very frequent words (*the*, *this*, *they*, *than*, *then*, etc.) and a relatively small number of rare words (*theirselves, theirself, thereabouts* and *thereof* are the only hapax legomena found in Switchboard). The frequent words in this sublexicon are also far more frequent than the frequent words in the flap sublexicon. In these respects, it is representative of a change that affects or is triggered by an unproductive sublexical unit, and therefore can be seen to lie on the opposite end of the continuum of productivity from the flap sublexicon (Baayen, 1993; Bybee, 1995). In principle, any other lexicon can be substituted: the predictions below are a necessary consequence of hierarchical inference and a highly skewed frequency distribution.

The log odds of reduction were seeded as in (1), with *b*_0_ set to either –1 or –3 on the logit scale in the simulations below (0.27 or 0.05 on the probability scale), the magnitude of the frequency effect *b*_Freq_ set to 0.02 or 0.0002. The effects of these manipulations are discussed below, but it is worth noting that the values allow the change to progress slowly enough for lexical diffusion to be observed, and to progress rather than sputtering out. A substantially higher *b*_Freq_ can make almost all words have ceiling rates of reduction, while a substantially lower one can make them all reduce at the same rate. A substantially lower *b*_0_ can lead the change to sputter out rather than progressing, and a higher *b*_0_ means that the change has already affected most of the lexicon. The random effect of word was set as a random distribution with a mean of 0 and standard deviation of 0.4. I have tried reducing the latter to 0.2 and increasing to 0.8 with little effect. The random effect of word corresponds to whatever factors influence the likelihood of reducing a word that are not captured by the word’s frequency. The three numbers mentioned above are the free parameters of the model, but the qualitative predictions are unchanged across a range of possible values. The number of reduced and unreduced tokens for each word was then generated as a sample from the binomial distribution, as in (2), with probability of reduction (*p*_red_) defined as the inverse logit of the log odds, (1), and number of trials defined as the frequency of the word.

(1) *p_red_* = *logit*^−1^(*b*_0_ + *b_Freq_* × *Freq* + *N*(0, *b_w_*))
(2) *n_red_* ∝ *Binom*(*p_red_*, *Freq*).

**FIGURE 1:**
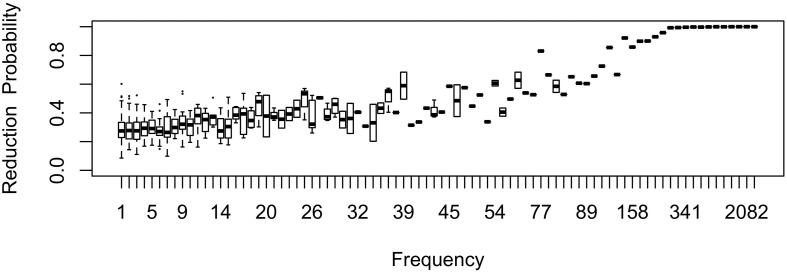
The effect of frequency in the first generation, prior to passing the language through inference. Note that the frequency axis is rank-transformed (with the highest frequency on the right). Boxes consisting only of the median line contain a single word.

The effect of word frequency in this first generation is illustrated in Figure 1 for *b*_0_ = –1 and *b*_Freq_ = 0.02. The shape of the effect in Generation 1 represents what one would expect the shape of the frequency effect to be if inference played no role in articulatorily-motivated sound change. As one might expect, the effect of frequency is monotonic, with greater reduction in frequent words. Because reduction in (1) is proportional to raw frequency, and the frequency distribution is Zipfian, reduction probability is much higher in the highest-frequency words than in the bulk of the lexicon: reduction is nearly categorical in the most frequent words, while the mean reduction probability is 32%, close to the expected probability for a word of zero frequency, *b*_0_ = 27%. Lowering *b*_0_ lowers the curve, lowering *b*_Freq_ reduces its slope, and lowering *b*_*w*_ (standard deviation) reduces the degree to which individual words deviate from the mean reduction probability at each point along the frequency axis.

Notice that the generative model in (1–2) is exactly that assumed by mixed-effects logistic regression with a by-word random intercept. Each generation was therefore assumed to use logistic regression to infer *b*_0_, *b_w_* and *b*_Freq_ or some subset thereof (Table 1). The regression was implemented using the lme4 package for R (Bates et al., 2015).^1^

**TABLE 1:** The model versions explored in the present paper.

Learner estimates	Reduction is influenced by	Section	Figures
*b*_0_, *b*_w_	Raw frequency	3.1	2, 3, 5, 7
	Log frequency	3.2	9, 10
*b*_w_	Raw frequency	3.1	4, 6
*b*_0_, *b*_w_, *b*_Freq_	Raw frequency	3.3	11

Each generation then regenerated the corpus. In the model version that did not infer an effect of frequency (top two rows in Table 1), the inferred random effects of words replaced *N*(0,*b*_*w*_) in (1), and the inferred fixed-effects intercept replaced *b*_0_ while the original *b*_Freq_ was retained. This represents the assumption that the effect of word frequency is due entirely to articulatory automatization. In the model that did infer the frequency effect (bottom row in Table 1), *b*_Freq_ was the sum of the inferred *b*_Freq_ and the original *b*_Freq_. This corresponds to the possibility that words can be reduced either because reduction is inferred to be appropriate in this context, or because of articulatory automatization.

The language passed through up to 20 (or 100 or 300, where noted) generations. Iteration was stopped early if average probability of reduction across the tokens of the regenerated corpus exceeded 99% or fell under 1%, which defined the change running to completion or sputtering out, respectively.^2^ 100 replications of the iterated learning process were performed for each parameter setting.

As mentioned above, the hierarchical structure assumed here is intended to be the simplest possible structure that can illustrate the effects of hierarchical inference on sound change. Additional influences on pronunciation can be easily incorporated into the model as additional fixed or random effects in Equation (1) above. For example, words can be nested within phonological contexts or morphemes to capture the fact that some morphemes can favor reduction across words, e.g., *-ado* favors Spanish intervocalic stop lenition (Bybee, 2002). Utterances or word senses can be nested in words to capture the fact that some uses of a word are more likely to be reduced. For example, *don’t* is more likely to be reduced in *I don’t know* than in *I don’t think* and especially if *I don’t know* is used to indicate uncertainty (Bybee and Scheibman, 1999). English auxiliaries are more likely to be reduced in some syntactic constructions than in others (Barth and Kapatsinski, 2017). Speakers (nested in social groups) can also be added as an additional random effect crossed with words, to implement inference of who flaps and who doesn’t. Interactions between random effects can also be added, e.g., to capture knowledge of differences in the effect of phonological context effects on t/d deletion across English dialects (Coetzee and Pater, 2011).

## Simulation Results

### Inference of a Random Effect of Lexical Identity: Lexicalization, Polarization, Stable Variation and a U-Shaped Frequency Effect

By treating lexical identity as a random effect, the model sidesteps the problem of estimating the effects of individual rare words, assuming that they will behave approximately like the average word, i.e., their reduction probabilities are drawn toward the mean reduction probability across all words. Partial pooling is of course necessary for the rarest of the rare, the words that the speaker has never before encountered, because the model has no information about whether a novel word favors or disfavors reduction. However, it is also rational for more frequent words: the speaker would have considerable uncertainty about the acceptability of a flap in a word s/he observed two or three times if s/he could not use information about the acceptability of the flap in other (similar) contexts to make this determination (Gelman and Hill, 2007: 252–259).

Treating lexical identity as a random effect means that the regression model performs partial pooling of the information about variant probability across words, optimally weighting information from tokens of the word against information from tokens of the same sublexical unit occurring in other words (Gelman and Hill, 2007: 252–259). In partial pooling, the extent to which a word is drawn to the mean is inversely proportional to its frequency. The less frequent a word, the less information we have about the effect of that word on pronunciation (or on anything else). Thus, to know how an infrequent word behaves, a rational learner will partially rely on information about the behavior of other (similar) words. In contrast, to know how a frequent word behaves it is not necessary to rely on information about the behavior of other words: tokens of a word are more relevant for inferring its behavior than tokens of other words, and so should be relied on to the extent that they are available in sufficient quantities to draw a reliable inference.

**FIGURE 2:**
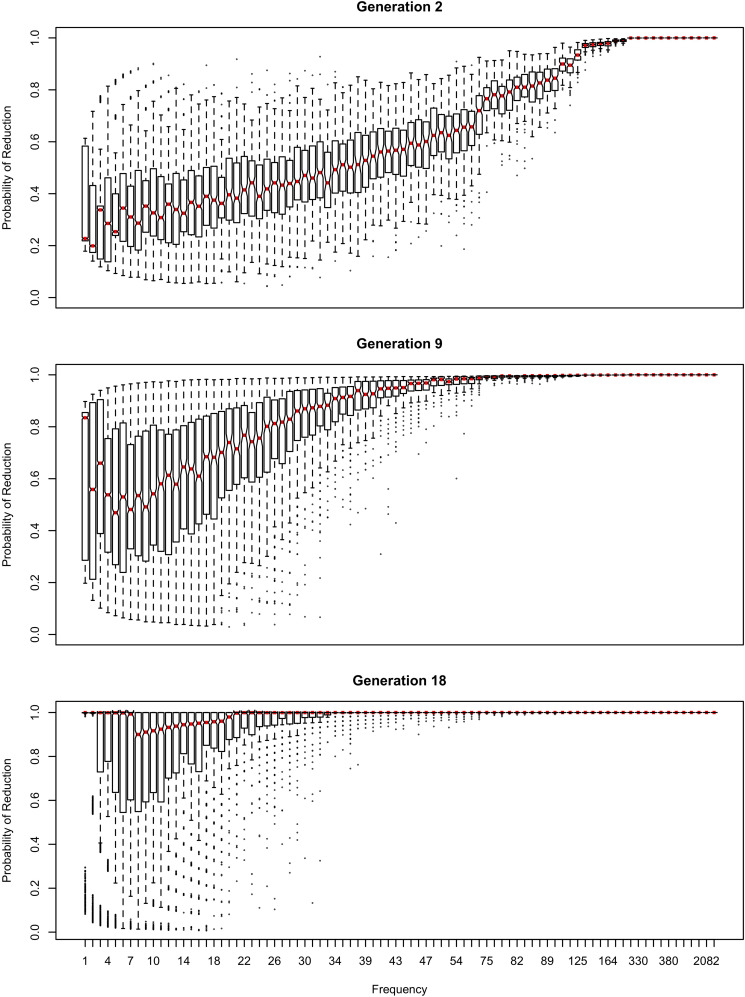
The effect of frequency if the learner estimates overall probability of reduction (*b*_0_) and the random effect of word, but not the effect of frequency across generations. Thick red lines show median probability of reduction at each frequency. Notches show the 95% confidence interval for the median.

**FIGURE 3:**
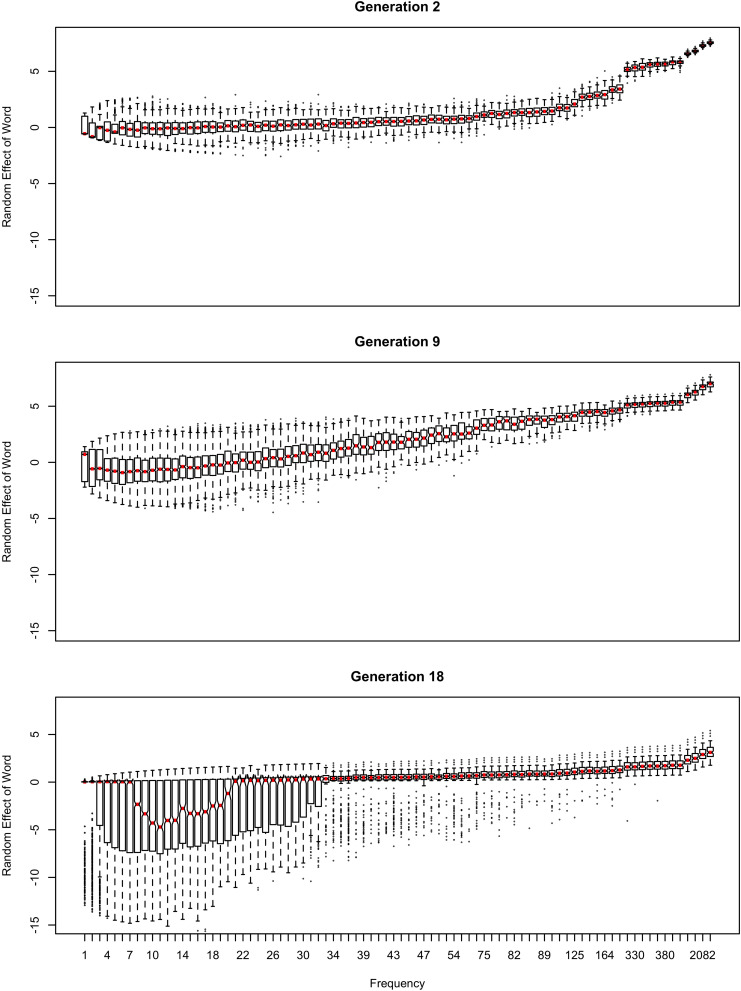
The random effect of lexical identity across generations. A negative random effect for a word indicates that the word is associated with the conservative variant. A positive one indicates that the word is associated with the innovative variant.

**FIGURE 4:**
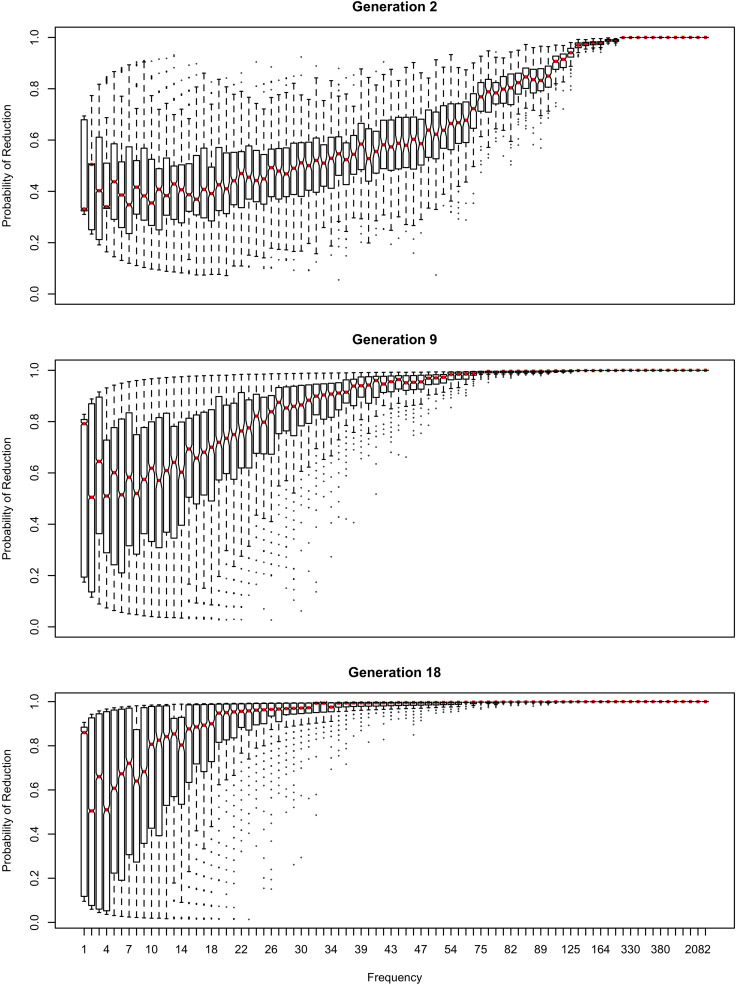
The effect of frequency if the learner estimates only the random effect of word across generations.

The influence of inference on the word frequency effect is shown in Figures 2–4. The top panel shows the effect of frequency after the first pass through the inference process (Generation 1). At this point, the reduced variant is in the minority, and therefore the frequency effect is always monotonic, greater frequency favoring reduction. The middle panel shows a generation for which the reduced variant has become the majority variant, but has not yet achieved dominance: for this generation, the reduced variant accounts for 60–70% of tokens. The bottom panel shows a generation for which the reduced variant is statistically dominant, accounting for 90% of tokens.

In Figure 2, the learner estimates *b*_0_, an overall probability of the reduced/innovative variant and the random effect of word on the choice, but does not estimate *b*_Freq_, the effect of frequency. Reduction results only from use / automatization of production, increasing with raw frequency as in (1–2). A pronounced U develops in the shape of the frequency effect (as shown in the middle and bottom panels of Figure 2). By Generation 9 (middle panel), the median reduction probability for hapax legomena (frequency = 1) is much higher than for words that are more frequent. By Generation 18 (bottom panel), the words with frequencies below 8 or above 20 are almost always reduced, but the median reduction probability is at 90–95% for words of intermediate frequencies. As shown in Figure 3, this U is caused by the random effect of word, which maintains a set of exceptionally conservative words. These words must be frequent enough for their effect on reduction probability to be reliably estimable, but not so frequent as to become reduced through automatization of articulation.

The change in this model tends to stall at around 91% reduction (bottom panels of Figures 2, 3). That is, the model gradually converges on nearly categorical use of the reduced variant, but the rate of change slows down dramatically once the probability of the reduced variant exceeds 90%. An individual chain can persist in the state shown in the bottom panels of Figures 2, 3 for a hundred generations. Furthermore, increasing or decreasing the size of the frequency effect by an order of magnitude changes how fast the model converges on ∼91% reduction but does not appear to help the model achieve greater reduction probability.

Table 2 shows the overall distribution of reduction probabilities across word types at Generation 100. The distribution shows what Zuraw (2016) has called *polarized variation*, which is characteristic of changes that have become lexicalized: the distribution of choice probabilities across words is highly bimodal, with clear peaks at 0 and 100%. A small number of words show intermediate behavior, with the vast majority of words (678.55 on average) always occurring with the reduced variant.

**TABLE 2:** The distribution of reduction probabilities in word types across 100 chains at generation 100.

0	0.01	0.1–0.2	0.21–0.3	0.31–0.4	0.41–0.5	0.51–0.6	0.61–0.7	0.71–0.8	0.81–0.9	1
6311	416	8	16	20	4	27	8	8	2	67855

*There are no words with reduction probabilities between 0.01 and 0.13, or between 0.89 and 1.*

About 10% of the words (63.11 on average) become exceptionally conservative, reducing 0% of the time, with 4.16 more words reducing with a 1% probability. These are the outlier points at the bottom of the probability scale in the bottom panel of Figure 2. These rare reductions occurs because reduction can result from either inference that the word should be produced with the reduced variant, or from automatization of production. The automatization of production is blind to lexical idiosyncracies, and is always pushing words to reduce. However, inference resists this push for words that are inferred to be conservative.

As shown in Figure 3, emergence and persistence of polarized variation happens because the model learns of a set of exceptionally conservative medium frequency words (bottom panel). When most words are reduced 100% of the time, their random effects are essentially zero. However, exceptionally conservative words are maintained because their random effects are strongly negative. As long as these exception words are frequent enough, it appears that they can be maintained indefinitely.

Even though change in this model is driven entirely by frequency of use, the correlation between frequency and reduction probability weakens dramatically over time. Thus, log frequency accounts for 27% of the variance in reduction probability at Generation 2, but only 8% by Generation 9, and essentially 0 variance by Generation 18 (0.02%). Thus, the effect of word frequency in this model is expected to weaken dramatically as an articulatorily-motivated change progresses. Some support for this prediction can be found in Cohen-Goldberg (2015), who found an effect of lexical frequency on /r/ deletion in a largely rhotic variety but not in a largely non-rhotic one. Furthermore, findings of weak or non-significant frequency effects in advanced changes (e.g., American English flapping in Warner and Tucker, 2011) are to be expected under this model, and do not provide evidence against articulatorily-motivated sound change being led by high-frequency words.

**FIGURE 5:**
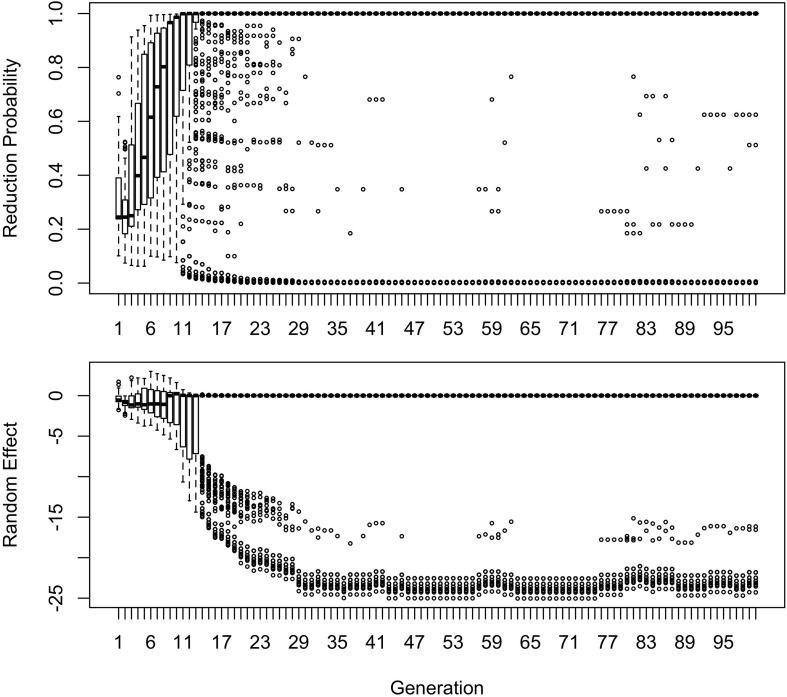
Reduction probabilities and random effects of words that had below-average reduction probabilities at Generation 2. One run of the model shown. In this model, “the middle doesn’t hold”, and variation becomes polarized, with individual words reducing or not reducing close to 100% of the time (top panel). The bottom panel shows that words favoring reduction favor it because reduction is probable in the lexicon as a whole. The words that disfavor it instead become “radicalized”, developing very strong negative random effects in favor of the conservative variant.

In Figures 4, 5, the learner estimates only a random effect of word, and does not estimate either the effect of frequency on choice (*b*_Freq_), or the overall probability of reduction (*b*_0_). This version of the model behaves like the model in Figures 2, 3 in developing a U-shaped frequency effect because the words are still implicitly grouped together through partial pooling, resulting in the rare words being pulled toward the mean for the lexicon. However, the pace of change is slower, and the model does not converge to strongly favor the reduced variant. Instead, the model oscillates around a 61–64% reduction probability with the frequency effect illustrated in the bottom panel of Figure 4 for at least 100 generations.

Thus, this model predicts that stable polarized variation will eventually develop, and that an initially phonetic change will become lexicalized, a trajectory that Bybee (2001) has argued to be a diachronic universal (see also Janda, 2003). The level at which the change stalls depends on whether the learner estimates an overall probability for a variant that is independent of individual words (*b*_0_); in other words, estimating which variant is more likely overall, or if s/he only estimates how variant choice is conditioned by context. This seems intuitively satisfying for known cases of stable variation, such as the choice between *-ing* and *-in’* in English progressives, where the choice is invariably strongly conditioned by contextual factors such as register (see Gardiner and Nagy, 2017, for a review). In the present simulations, the conditioning contexts are lexical, thus the change becomes lexicalized, but other conditioning variables can be easily added to the model to investigate how a variant can become restricted to other environments, like speech styles or social personae.

The initial random intercepts with which words are seeded are not strong enough to resist reduction after the innovative variant becomes the default. How then do exceptionally conservative words become exceptionally conservative, enabling the conservative pronunciation to survive indefinitely? The bottom panel of Figure 5 shows the random effect of word across the generations in one run of the model from Figure 2. In this model, when the innovative variant becomes dominant, most words succumb to the analogical pressure to reduce, regressing to the mean of the lexicon. However, a minority of words have random intercepts that are low enough for them not to regress to the mean of the lexicon at this point. These words then become “radicalized”: the random intercepts of these words become ever more extremely negative in order to account for their lack of reduction. This makes these words increasingly resistant to reduction, stabilizing the system. Essentially, as the likelihood of reduction increases, the learner becomes increasingly confident that there is something special about the words not affected by reduction that prevents it from affecting them, resulting in lexicalization of the sound change.

Radicalization also happens in the model without an overall intercept shown in Figure 4, although here it is less extreme and affects both innovative and conservative words. Because all variability must be attributed to lexical identity, reduction caused by automatization of production leads to an increase in the corresponding random intercepts. The random intercepts of conservative words then must decrease to account for them now being farther from the lexicon mean. Because there is no overall intercept favoring the reduced variant across words, analogical pressure to regress to the mean is weaker, and variation stabilizes at a less skewed distribution. Interestingly, this distribution is also somewhat less polarized, with modes at 0.05 instead of zero and both at 0.95 and 1. Nonetheless, the variation remains stable after Generation 20.

**FIGURE 6:**
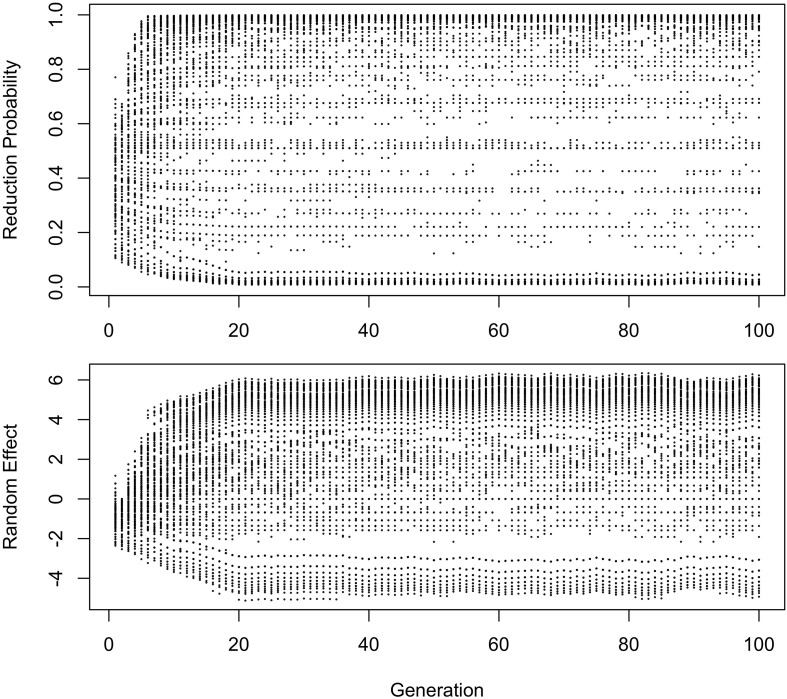
Changes in the distribution of reduction probabilities and random effects for words with below-average reduction probability at Generation 2 in the model without an overall intercept. Negative random effects are in favor of the conservative variant.

**FIGURE 7:**
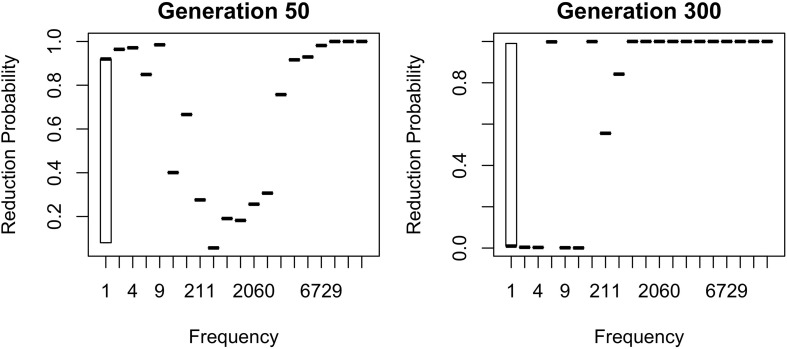
Two generations with a similar mean probability of reduction of the /ð/ sublexicon. Note. *b*_Freq_ was reduced to 0.0002 for this simulation from 0.02 in Figures 1–6 and *b*_0_ was reduced to –3 from –1. This causes the model to converge more slowly, but the results are qualitatively similar if these parameters are higher.

The results in Figures 1–6 replicate on a different lexicon, the set of English words that start with /ð/. As mentioned above, this sublexicon is representative of a change that affects or is triggered by an unproductive sublexical unit, and therefore can be seen to lie on the opposite end of the continuum of productivity from the set of words examined in Figures 1–6 (Baayen, 1993; Bybee, 1995). Nonetheless, the results in this dataset are qualitatively very similar to those above: a U-shaped frequency effect develops as the reduced variant becomes dominant (Figure 7), and the change stalls as it becomes lexicalized, because exceptionally conservative words become radicalized when the reduced variant comes to dominate the lexicon. Thus, I expect these predictions to hold across a wide range of naturalistic sublexica eligible to undergo a particular sound change.

As with the flap sublexicon, not estimating an overall probability of reduction results in settling on a lower reduction probability. Interestingly, the overall reduction probability stabilizes much longer than individual words do. Thus, although probability of the reduced variant fluctuates around 0.67 for a long time, this stability initially masks large changes in the behavior of individual words from generation to generation as automatization-driven reduction battles entrenchment in conservatism for frequent words. Specifically, the mean reduction probability is about the same in both panels of Figure 7 (0.65 on the left, 0.68 on the right) but the state represented in the left panel of Figure 7 is unstable, and the model eventually converges to the state resembling the right panel, with all frequent words being categorically reduced.

### Stable Variation Depends on the Frequency Distribution and Its Effect on Reduction

The behavior of the model is dependent on the assumption that Equation (1) uses raw frequency and not log frequency. One might object to this assumption because log frequency is observed to be a better linear predictor of many behavioral dependent variables (e.g., Kapatsinski, 2010a, for error rate; Oldfield and Wingfield, 1965, for production latency). However, interestingly, this superior fit of log frequency turns out to also be true in the data generated by (1–2), even though they were generated using raw frequency: log frequency captures 27% of the variance in the generated reduction probabilities at Generation 1, compared to 18% captured by raw frequency. Thus, log frequency can fit the data better than raw frequency even if the data are generated by a model that is sensitive to raw frequency, i.e., a system in which every token of experience matters equally (as argued by Heathcote et al., 2000, for the effects of practice in general). This happens because there is an upper limit on reduction probability, so it always looks like the effect of frequency on reduction decelerates as reduction probability approaches the upper limit.

**FIGURE 8:**
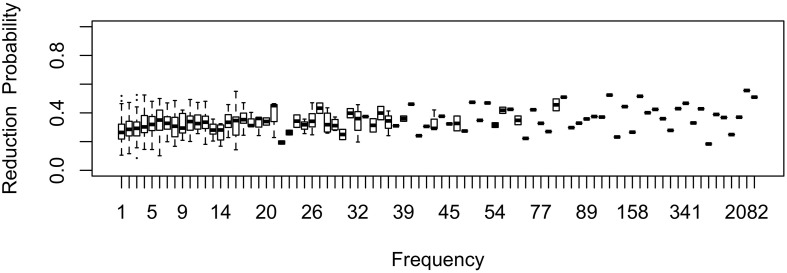
Effect of frequency on reduction probability for Generation 1 (before the language is subjected to inference) with reduction driven by log frequency in Equation (1). *b*_Freq_ = 0.11 rather than 0.02, so that mean *b*_Freq_×*Freq* in Figure 1 is the same as mean *b*_Freq_×log(*Freq*) here.

**FIGURE 9:**
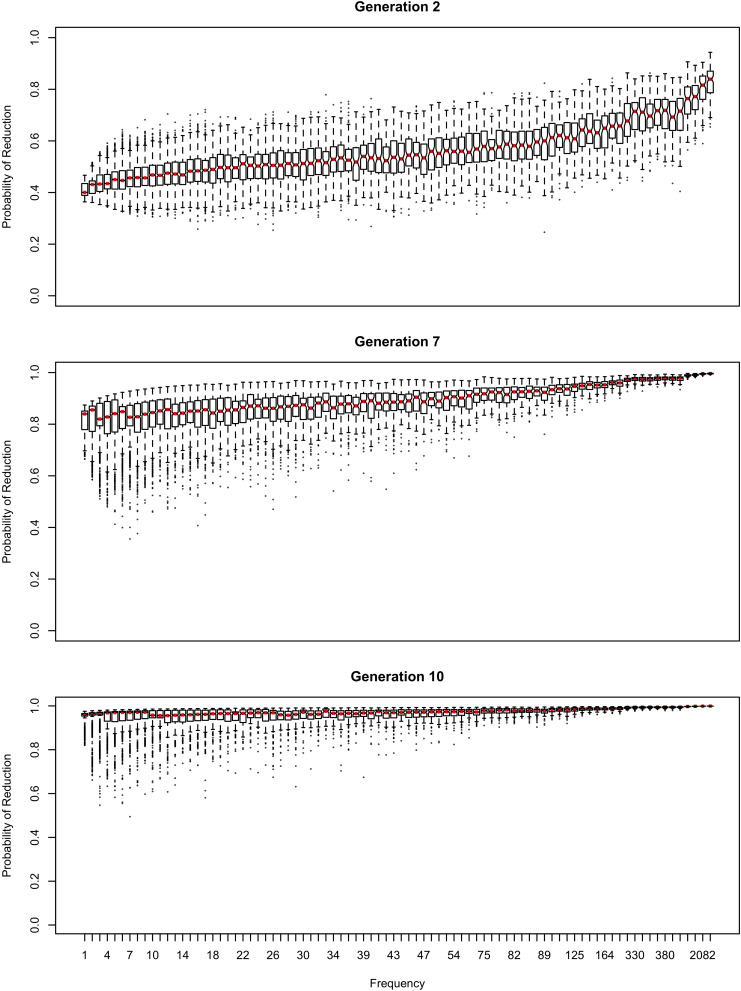
The effect of word frequency if reduction is driven by log frequency. The learner does not estimate the effect of word frequency in this simulation.

If log frequency is used in Equation (1), as illustrated in Figure 8 (cf., Figure 1), the sound change progresses more quickly (Figure 9), even if mean *b*_Freq_×*Freq* is equal to mean *b*_Freq_×log(*Freq*). As mentioned earlier, mean reduction probability is in the 81-91% range across replications by Generation 20 with raw frequency, and can persist in that range for a hundred generations. In contrast, sound change completes at Generation 13–14 when reduction is driven by log frequency, even though it looks less advanced prior to training (Figure 8 vs. Figure 1). This is due to the Zipfian distribution of word frequencies. With raw frequency driving reduction, the reduced words form a small minority for a long time: for a randomly chosen word type, the reduction probability is almost as low as that of a novel word. Therefore, the overall probability of reduction grows slowly. This allows some words time to become entrenched in their conservatism: they coexist with highly reduced frequent words. Furthermore, the frequent words in Figure 1 are clear outliers relative to the mean of the lexicon. Their behavior is due to frequency but the learner does not know this, and therefore attributes it to a random effect of word. As result, the learner comes to believe that words have substantial idiosyncrasy: it is possible for a word to be really far from the lexicon mean in their reduction probability (as far as 5 standard deviations above in the top panel in Figure 3).

**FIGURE 10:**
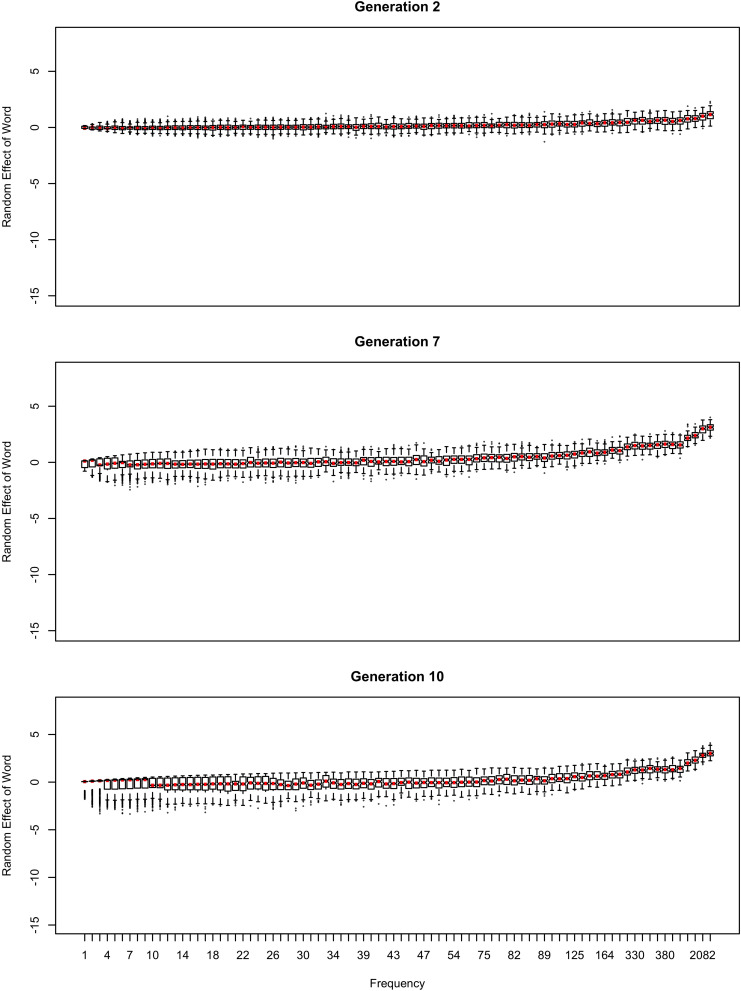
Random effects in the model if reduction is driven by log frequency in Equation (1).

Because word is treated as a random effect, the learner estimates how variable the population of words is. Because of partial pooling, outlier words regress to the average reduction probability across words to the extent that words in general are tightly clustered around the average reduction probability. Therefore, estimating that words are highly variable in reduction probability allows exceptionally conservative words to not converge to the lexicon mean (Figures 3, 5), which is what allows the conservative pronunciations to then be replicated across generations indefinitely. If reduction is proportional to log frequency, random effects are not so extreme: words look much more alike to the learner (Figure 10, which is on the same scale as Figure 3), hence sound change can run to completion relatively easily.

In addition, the U-shape predicted to occur in the later stages of a sound change is reduced, though not eliminated, and occurs at a higher average reduction probability than if raw frequency is used in (1). Nonetheless, the qualitative prediction is the same: once the reduced variant comes to dominate the lexicon, novel words should be reduced more than conservative medium-frequency words.

The difference between the initial distributions in Figures 1, 8 is that, in Figure 1, there are words whose behavior strongly deviates from that of the majority. This deviation is due to their higher frequency, coupled with the shape of the effect of frequency on reduction and the Zipfian distribution of word frequencies. Because their raw frequency is dramatically higher than that of the average word, and reduction rate tracks raw frequency, the frequent words look exceptional to a learner that cannot conceive of frequency as an explanation for these words’ high degree of reduction.

That is, polarized variation requires the sublexicon of words affected by a change to contain apparently exceptional words. Although the first apparently exceptional words are exceptionally innovative, and these words become unexceptional as the lexicon approaches their reduction rates over generations, their existence is what allows for exceptionally conservative words to emerge and persist. This leads to a rather counterintuitive prediction: removing the highest-frequency, most reduced words from the sublexicon affected by a sound change should allow the sound change to run to completion even if the effect of frequency on reduction tracks raw frequency. This prediction is counterintuitive because this change makes the initial average probability of reduction lower. I have confirmed this prediction by creating an artificial version of the /ð/ sublexicon by removing words from the head of the frequency distribution (creating a ‘headless’ distribution; Harmon et al., 2021). Specifically, I removed all words with a frequency above 900 tokens in Switchboard, leaving only the 3 hapax legomena and 7 more frequent words (with frequencies 3, 4, 7, 9, 20, 30, and 211 tokens). This type of distribution might characterize a rare sound that occurs only in a small set of borrowed words (which are likely to be infrequent), such as the /ʒ/ word onset exemplified by *genre*. Even though removing the head of the distribution reduces the initial probability of the innovative variant, it allows the change to run to completion, with the innovative variant eventually dominating the production of all words. In other words, a change that affects a sublexicon of words of similar frequency is more likely to run to completion than a change that affects a sublexicon of words whose frequencies are very diverse. On the other hand, the change is also more likely to sputter out, with all words converging to the conservative variant. What does not frequently happen is a state of stable polarized variation (Table 3, left column), although two chains did converge on reduction in the most frequent word and lack of reduction elsewhere.

**TABLE 3:** The effect of frequency on reduction, and the outcome of change.

Outcome of change	Weak reduction (*b*_Freq_ = 0.0002)	Strong reduction (*b*_Freq_ = 0.02)
Sputtered out	65	0
Run to completion	33	2
Stable polarized variation	2	98

*b_0_ = –3, headless /ð/ lexicon, learners estimate b_0_ and a random effect of word.*

Similarly, a change is more likely to run to completion if the size of the effect of practice on reduction is small, because the small effect size ensures that no words are inferred to be exceptional. That is, articulatorily-motivated changes to segments that are less likely to change as a result of practice paradoxically have a greater chance of running to completion (although they also have a greater chance of sputtering out). With *b*_Freq_ = 0.02, the headless /ð/ sublexicon tends to quickly become lexicalized because the more frequent words are reduced much more than the less frequent words (Table 3, right column), even though the change frequently runs to completion with *b*_Freq_ = 0.0002 (Table 3, left column); a significant difference, *p* < 0.0001 (Fisher exact test). Because the initial probability of reduction is low (0.05), the final stable state tends to involve either 2 or 3 most frequent words categorically adopting the innovative variant, with the rest being categorically conservative (24 and 44 chains, respectively). However, occasionally the innovative variant spreads to most words, with a couple medium-frequency holdouts (4 chains), and sometimes even runs to completion (2 chains).

The results are similar with the larger flap lexicon, but differences in outcome between chains are smaller because the lexicon is larger, thus estimates of reduction probability are more stable and less affected by the exclusion of the few high-frequency words. In particular, strong reduction in the headless flap lexicon restricted to have the same maximum token frequency as the headless /ð/ lexicon always converges on stable polarized variation, but the final probability of reduction is much less variable, falling within 0.04 of 0.22. Weak reduction can still both sputter out or run to completion but the pace of change is much slower than in the /ð/ lexicon.

### If Novel Words Are Thought to Be Like Rare Words, Frequency Effect Will Stay Monotonic

In all simulations reported above, a U is predicted to emerge in the shape of the frequency effect when the innovative, reduced variant becomes the default for the sublexicon. At that point, novel words would enter the lexicon with the reduced variant, while existing exceptionally conservative words would still be produced with the conservative variant.

**FIGURE 11:**
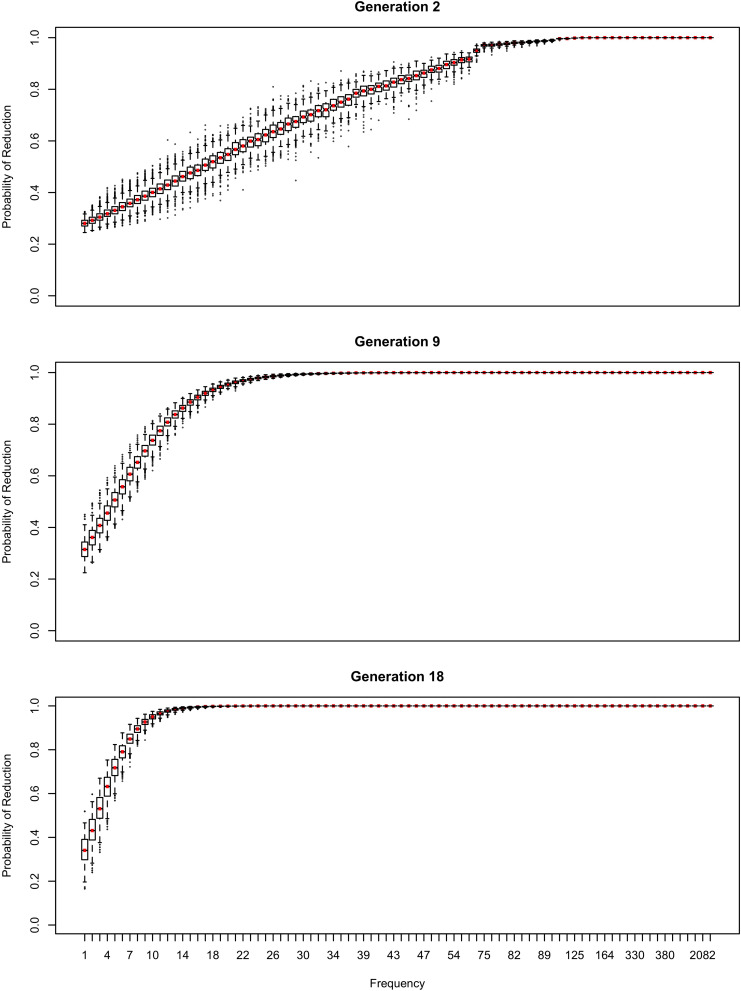
The effect of frequency over time if the learner estimates the influence of frequency on variant choice as well as an overall probability of variant and the random effect of word.

As shown in Figure 11 for the flap sublexicon, this prediction does not arise if the learner estimate the effect of frequency on variant choice, thus estimating all three *b* parameters in (1). In this model, the choice of the reduced variant can result either from the speaker’s belief that this variant is more appropriate / likely, or from use / automatization of production: for every generation after the first one, the inferred *b*_Freq_ is added to the original *b*_Freq_. As can be seen in Figure 11, no U-shape develops: the effect of frequency remains monotonic through the generations. The results in Figure 9 do not change substantially if log frequency is used instead of raw frequency in Equation (1).

If the effect of frequency is estimated, the likelihood of the change running to completion is strongly dependent on the size of the frequency effect (*b*_Freq_): with a strong reduction pressure (e.g., 0.1), the change runs to completion regardless of other parameters. However, with a weaker effect (e.g., 0.02), change does not run to completion. The change settles into stable variation at a reduction probability that depends on whether the learner estimates an overall intercept (*b*_0_, the probability of variant choice). If they don’t, the final reduction probability is quite high (above 90% in the flap sublexicon). If they do, then individual chains of learners estimating both *b*_Freq_ and *b*_0_ settle on oscillating around ∼55% of innovative variant choice with the same parameter setting (*b*_Freq_ = 0.02). Indeed, average probability of reduction is able to progress beyond the initial state in Figure 1 at all in this model only because of the additional reduction that comes from the incrementation of reduction probability by automatization of articulation: if only the inferred *b*_Freq_ is used, or the inferred and original *b*_Freq_ are averaged, the overall probability of the innovative variant does not increase across generations.

Variation in this model is not polarized: there is little variation in reduction probability between words of the same frequency; indeed, the random lexical variation the model is seeded with (Figure 1) reduces over time (Figure 11). Instead, stability comes from the model settling into a state in which only the lowest-frequency words (hapax legomena) are relatively unlikely to be reduced. The state to which this model converges if it does not estimate *b*_0_ is similar to that shown by flapping in American English: there are no known words in which it is categorically impermissible, it occurs > 90% of the time, existing words reduce at similar rates across most of the frequency range, but novel words or words are produced with a full stop more often than known words (Herd et al., 2010; Warner and Tucker, 2011). The present model suggests that variation does not become polarized if differences in reduction rates across words are attributed to something other than their lexical identity. A rational learner that attributes the differences in reduction probabilities between frequent and infrequent words to frequency does not attribute this difference to lexical identity: frequency explains away apparent lexical idiosyncrasy. The model in Figure 9 attributes them to frequency, but this is of course not the only possible factor conditioning variant choice. More generally, inference predicts that lexicalization should not happen when there are clear conditioning factors that account for between-word differences, whether these factors are social, stylistic, language-internal, or (like the effect of frequency) experiential.

## Discussion

This paper has examined the consequences of assuming that rational probabilistic inference is involved even in sound changes that are driven by automatization of production. Unlike analogical changes, these are sound changes that affect frequent words first (Schuchardt, 1885; Fidelholtz, 1975; Hooper, 1976; Phillips, 1984, 2001; Mowrey and Pagliuca, 1995; Bybee, 2001). In usage-based work, such changes have been discussed as resulting from automatization of holistic production plans associated with frequent words and collocations (Mowrey and Pagliuca, 1995). However, this hypothesis did not account for the fact that certain articulations are more likely to be affected by reduction than others, in a way that is specific to a particular language variety (e.g., Cohen Priva, 2017). To account for this property of change, Pierrehumbert (2002) proposed that articulatorily-motivated sound change affects sublexical articulatory units tagged with the larger lexical contexts in which they occur. The present model builds on this insight by allowing the learner to optimally allocate credit for an observed pronunciation between a segment and the larger context using hierarchical inference. In this paper, I examined how the predicted trajectories and outcomes of articulatorily-motivated sound change are affected by the assumption that the first language learner engages in this type of inference.

Sound change is commonly seen to result in a pattern of stable, lexicalized variation in which some words remain exceptionally conservative (e.g., Bybee, 2001). Zuraw (2016) points out that lexicalization results in a pattern of polarized variation, where some words occur with one pronunciation variant 100% of the time or nearly so, and others (almost) never occur with the variant. A model of articulatory optimization that does not provide a role for inference predicts that an articulatorily-motivated sound change will ultimately affect all words as their productions are optimized over generations. Hierarchical inference explains why changes might stall, and how polarized variation arises. Specifically, polarized variation occurs if articulatory reduction affects different words at very different rates, and the learner attributes these differences to lexical identity rather than their true cause. Here, that true cause is simple frequency of use, but it could also be occurrence in reduction-favoring linguistic or social contexts (as in Bybee, 2002, 2017; Brown, 2004; Raymond and Brown, 2012). An important direction for future work is to differentiate between frequency of occurrence in reduction-favoring vs. disfavoring contexts. The literature is ambiguous regarding whether occurrence in reduction-disfavoring (e.g., formal) contexts merely delays change, or can actually lead the change to reverse direction. That is, it is not yet clear whether an additional token of occurrence in a reduction-disfavoring context, should decrement the probability of using the reduced variant in other contexts. It would be interesting to examine the consequences of this assumption.

Polarized variation arises through radicalization of exceptionally conservative words. Radicalization occurs because of the co-existence of conservative words with exceptionally innovative words in earlier generations, which leads the learner to estimate a large random effect of word. As the innovative pronunciation spreads through the lexicon, previously innovative words become the new mainstream, but their prior exceptionality allows exceptionally conservative words to retain their conservative pronunciations. That is, exceptions beget exceptions, even though the composition of the set of exceptions changes radically over time.

Hierarchical inference predicts that an articulatorily-motivated change can sputter out. Without this mechanism, articulatorily-motivated change inexorably marches on through the lexicon, converging to the reduced variant. However, in real life, the same change can sometimes take off, and sometimes not. In their foundational monograph on language variation and change, Weinreich et al. (1968) called this the actuation problem, and suggested that the answer to it is to be found in social dynamics – how an incipient change diffuses through society. The present simulations suggest that actuation also depends on lexical diffusion of the change: depending on the frequency distribution in the sublexicon of words that contain the structure affected by the change, and how the words that tend to occur in reduction-favoring contexts are distributed over the frequency spectrum, a change may not take off. In particular, if the effect of practice on the articulation is relatively weak for the sound in question, the sublexicon affected by the change happens to contain few high-frequency words (which are the words strongly affected by the reductive effect of practice), and the innovative pronunciation variant is initially rare, the change often sputters out. I submit that sputtering out is how changes ‘do not happen:’ variants that spread and take over in other languages arise and then disappear because they are inferred to have a low production probability. In essence, the speaker guards against reductions that they consider to be errors, suppressing their production. Covert error monitoring and suppression is of course well known to occur in language production (Motley et al., 1982). The present model shows one diachronic trajectory by which errors come to be seen as errors. Of course, there is always a chance for one of these variants to arise again because automatization of production continues to favor it over the conventionalized conservative alternative.

What can influence the strength of the influence of practice on articulation (*b*_Freq_)? The most obvious influence on this parameter is the fact that certain articulations are easier to produce in the context in which they occur than others. Articulations would not be particularly subject to the effect of practice. However, some articulations may also not change much as a result of practice even though they are not easy to articulate in context. For example, the tongue blade is a relatively fast, light and (at least for an adult) easily controllable articulator. It therefore appears relatively easy to speed up the production of a blade-raising gesture during the production of an alveolar stop with practice, turning it into a flap. In contrast, the tongue body is slow and heavy, making it much harder to speed up the production of a velar stop. Quantal effects, where certain articulatory changes lead to large changes in acoustics and other articulatory changes of the same magnitude do not (Stevens, 1989), can also make certain articulations more malleable due to absence of corrective feedback from interlocutors or the speaker’s own perceptual system.

What can influence the initial probability of reduction (*b*_0_)? It seems likely that some changes originate from selection of variants that fall within a range of acceptable articulations before the change happens (Blevins, 2004). For example, there is a wide range of acceptable palatal constriction magnitudes for a Spanish [j]∼[ʒ], and selection of variants from within this range can drive divergence between dialects (Harris and Kaisse, 1999). Tongue positioning during a vowel is also quite variable, as is constriction magnitude in the production of an English flap (De Jong, 1998). In contrast, other changes might originate in speech errors, which may initially be very rare. A possible example is [θ] > [f] (Honeybone, 2016), because [f] and [θ] are not part of a continuous articulatory range of variants. In addition to changes that are not within an articulatory range associated with a production target, low initial production probability may hold for variants that are saliently perceptually different from the conservative variant, and therefore likely to be noticed by the listener (and perceived as a mismatch with intended acoustics by the speaker). Thus, changes that cross a quantal boundary might start out from a lower production probability. The simulations in the present paper show that such changes are likely to die out, but can also gain strength over time and even run to completion.

A take-home point of the present paper is that inference makes the dynamics of sound change rather chaotic; particularly so when the sublexical structure affected by the change has a low type frequency (like an initial /ð/ in English). Depending on small differences in initial conditions, and noise inherent to probabilistic selection of variants to produce, the same change affecting the same lexicon will sometimes go to completion, sometimes lexicalize, and sometimes sputter out. This is true in the present simulations even though there is no social environment to provide an additional source of variation. This means that the actuation problem is likely unsolvable. We should not expect to be able to predict whether a change will or will not happen. However, a theory of sound change can predict the directions in which change will proceed if it does, and a model that incorporates inference can help identify the factors that make a change more or less likely to be actuated, and to be lexicalized.

An intriguing prediction of hierarchical inference is that exceptionally conservative words should emerge in a ‘sweet spot’ in the frequency range when an articulatorily-motivated sound change enters a late stage in its development. When the reduced pronunciation becomes more likely, across the lexicon, than the original one, new words entering the lexicon should adopt the reduced pronunciation. Therefore, these new words should be more reduced than exceptionally conservative words. An important direction for future research is to model the impact of new words entering the lexicon on change. A limitation of the present implementation is that the lexicon is constant throughout. However, new words actually enter the lexicon all the time, and not at a constant rate (Gershkoff-Stowe and Smith, 1997). It would be interesting to see how the trajectory of change is influenced by state of the sublexicon when a large number of new words is encountered. An additional complication arises from the finding that words that have difficult articulations are especially likely to be replaced with other words because their articulation difficulty makes it less likely that they are selected for production (Berg, 1998; Martin, 2007).

Hierarchical inference predicts the effect of word frequency to be non-monotonic in the later stages of a reductive sound change. The most frequent words will be reduced because of two reasons: (1) the articulatory pressure toward reduction, as well as (2) because they were reduced in the input to the current generation of learners and thus will be associated with the reduced variant of the phone. The least frequent words will be reduced because they are not associated with any variant of the phone, and the reduced variant is more frequent. At intermediate frequency levels, some words, which happened to be often used with the unreduced variant of the phone by previous generations, can become associated with the unreduced pronunciation variant. As mentioned earlier, this prediction presupposes that a particular way of pronouncing a sublexical unit can spread from word to word, as suggested by Pierrehumbert (2002). This assumption is supported by the empirical results on new dialect acquisition in German et al. (2013), where speakers of American English were shown to rapidly learn new pronunciations for particular phones, e.g., a glottal stop in place of a flap, with no evidence of learning being restricted to individual words experienced during training (see also McQueen et al., 2006; Peperkamp and Dupoux, 2007; Maye et al., 2008; Nielsen, 2011).

An important contribution of the present simulations is to show the conditions under which exceptionally conservative words should emerge. This prediction of a U-shaped frequency effect in the later stages of an articulatorily-driven sound change is inevitable as long as (1) the sublexicon affected by the change includes frequent words that reduce at much higher rates than the rest of the sublexicon, and (2) the relationship between word frequency and variant choice is due solely to automatization of production, rather than to inference. That is, the learner should assume that novel words are likely to behave like the typical word, rather than like the typical *rare* word. This assumption is often made in research on productivity, because speakers tend to apply grammatical patterns to novel words based on the proportion of known words that obey them (see Kapatsinski, 2018b, for a review). However, Pierrehumbert and Granell (2018) found that the morphological behavior of hapax legomena is predicted by the behavior of rare words better than by the behavior of frequent words (see also Baayen, 1993; Zeldes, 2012; but cf. Albright and Hayes, 2003). Because productivity of a pattern is defined as its applicability to novel words, the particular importance of rare words in increasing productivity of a pattern suggests that learners infer the behavior of novel words from the behavior of (other) rare words, rather than from the entire lexicon. The question is whether speakers also implicitly know that the same phone (or letter) is likely to be pronounced differently in rare and frequent words, and make use of this knowledge in production.

It is also possible that speakers infer the likely pronunciations of words that they encounter more indirectly, by inferring the word’s provenance. For example, speakers often need to infer the linguistic origin of a word to know how to pronounce it ‘properly’. Relatedly, Vaughn and Kendall (2019) show that American English readers use the orthographic cue of an apostrophe at the end of a verb like *walkin’* to change their pronunciation of the rest of the utterance in a way that sounds more casual and Southern. For an adult native speaker’s extensive experience with the language, the fact that the word is novel suggests that it is the kind of word that occurs in contexts with which the speaker has had little experience. For the typical experimental participant, a native-speaker university student, most newly encountered likely come from formal, academic contexts. They may therefore infer a novel word to likely be of similar provenance and thus pronounce it in a more formal fashion.

An important direction for future research is to extend the model to continuous articulatory variability. In principle, nothing in the proposed model depends on categoricity of the choice. For example, although we model reduction as the choice of a discrete variant here, a U-shape should also emerge if it were treated as a continuous acoustic or articulatory parameter (such as duration or degree of closure for a stop/flap/approximant continuum). The U shape depends on treating word as a random effect, and would emerge whether the learner estimates a logistic regression model (as here) or a linear regression model, as would be appropriate for a continuous variable. Nonetheless, a categorical choice produces certain discretization of the probability space because a difference in choice probabilities is only observable when it corresponds to a difference in token counts. This makes extreme probabilities more likely to converge to zero and 1, especially in rare words (e.g., Kapatsinski, 2010a). Variation could therefore, perhaps, be less polarized if the speakers were estimating a continuous parameter that is faithfully represented in the signal.

## Conclusion

In this paper, I have explored the role of hierarchical probabilistic inference in articulatorily-motivated sound change, motivated by the findings that units at many levels of the linguistic hierarchy simultaneously influence pronunciation of a sound embedded in a particular context (Pierrehumbert, 2002). For example, pronouncing a /t/ as a flap in a particular phonological context could be due to the high probability of flapping in a favorable phonological context of a following unstressed vowel, or a high-frequency or informal word like *whatever*, which can lead to reduction outside of favorable phonological contexts (Shport et al., 2018). Because units at multiple levels (sublexical, lexical, and collocational) are jointly responsible for a particular pronunciation, a rational learner should allocate credit for a particular pronunciation across the levels via hierarchical inference. The proposed model provides a way to resolve the long-standing debate between proponents of regular sound change and proponents of lexical diffusion: it is not that “sounds change” or “words change”. It is both. Hierarchical inference provides a way to estimate the contribution of both sounds and words to particular pronunciations. The present model suggests that speakers make use of this power.

The proposed model therefore incorporates the following assumptions: (1) there are both words and sounds, (2) a word’s use causes reduction of the sounds in that word, and (3) both words and sounds (modeled as groups of words) are associated with reduction probabilities, with rational hierarchical inference adjudicating how much credit for a particular pronunciation of a sound in a word is assigned to the word vs. the sound.

The model explains how an articulatorily-motivated change can become lexicalized, even though there is a consistent pressure pushing all words to reduce. It also demonstrates the emergence of polarized variation (Zuraw, 2016). The model makes specific predictions about the circumstances under which a sound change can become lexicalized, the conditions under which it can sputter out, and the conditions under which it is likely to run to completion. Because chance plays an important role in determining the outcome of change, even in the absence of social influences, these predictions require a large-scale study of the characteristics of sublexica affected by changes that do and do not become lexicalized.

The hypothesis that speakers infer how to pronounce novel words based on generalization from a population of known words begs the question of what the relevant population is. Because rare words are often systematically different from frequent words (Bybee, 2001; Pierrehumbert and Granell, 2018), it can be considered rational for the learner to infer that a novel word will behave like other *rare* words, rather than being a typical representative of the whole sublexicon containing a sound eligible to undergo a change. When the innovative, reduced pronunciation becomes the majority variant, a learner who does not estimate the effect of frequency on pronunciation should favor the reduced pronunciation in novel words compared to known exceptionally conservative words. In contrast, a learner who does estimate the effect of frequency on variant choice should always show a monotonic frequency effect, with novel words being the least reduced. This provides another interesting direction for future empirical work.

## Data Availability Statement

The code for the model can be found in the Supplementary Material.

## Author Contributions

The author confirms being the sole contributor of this work and has approved it for publication.

## Conflict of Interest

The author declares that the research was conducted in the absence of any commercial or financial relationships that could be construed as a potential conflict of interest.

## Publisher’s Note

All claims expressed in this article are solely those of the authors and do not necessarily represent those of their affiliated organizations, or those of the publisher, the editors and the reviewers. Any product that may be evaluated in this article, or claim that may be made by its manufacturer, is not guaranteed or endorsed by the publisher.

## Footnotes

1 *glmer*(*variant* ∼ (1 | *w*), *family* = “*binomial*”). or, if the effect of frequency is estimated, *glmer*(*variant* ∼ (1 | *w*) + *Freq*, *family* = “*binomial*”). lme4 is used here because it converges relatively quickly, but informative prior beliefs can be added by replacing glmer() with brm() from the brms package (Bürkner, 2017) and specifying the desired prior.

2 The early stoppage rule was introduced because the logistic regression command produces an error, which stops the iteration over replications when the response is constant, i.e., sound change has run to completion.

## References

[B1] Abramowicz Ł (2007). Sociolinguistics meets exemplar theory: frequency and recency effects in (ing). *Univ. Pennsyl. Working Papers Ling.* 13:3.

[B2] Albright A. Hayes B. (2003). Rules vs. analogy in english past tenses: a computational / experimental study. *Cognition* 90 119–161. 10.1016/s0010-0277(03)00146-x 14599751

[B3] Baayen H. (1993). *On Frequency, Transparency and Productivity. In Yearbook of Morphology 1992.* Dordrecht: Springer, 181–208.

[B4] Babel M. (2012). Evidence for phonetic and social selectivity in spontaneous phonetic imitation. *J. Phonetics* 40 177–189. 10.1016/j.wocn.2011.09.001

[B5] Bannard C. Klinger J. Tomasello M. (2013). How selective are 3-year-olds in imitating novel linguistic material? *Dev. Psychol.* 49 2344–2356. 10.1037/a0032062 23458663

[B6] Barth D. Kapatsinski V. (2017). A multimodel inference approach to categorical variant choice: construction, priming and frequency effects on the choice between full and contracted forms of am, are and is. *Corpus Ling. Linguistic Theory* 13 203–260. 10.1515/cllt-2014-0022

[B7] Barth D. Kapatsinski V. (2018). “Evaluating logistic mixed-effects models of corpus-linguistic data in light of lexical diffusion,” in *Mixed Effects Models in Linguistics*, eds Speelman D. Heylen K. Geeraerts D. (Cham: Springer), 99–116. 10.1007/978-3-319-69830-4_6

[B8] Bates D. Maechler M. Bolker B. Walker S. (2015). *lme4: Linear Mixed-Effects Models Using Eigen and S4. R Package Version 1.0-4.*

[B9] Berg T. (1998). *Linguistic Structure and Change: An Explanation From Language Processing.* Oxford: Oxford University Press.

[B10] Browman C. P. Goldstein L. (1989). Articulatory gestures as phonological units. *Phonology* 6 201–251. 10.1017/s0952675700001019

[B11] Brown E. L. (2004). *The Reduction of Syllable -Initial /s/ in the Spanish of New Mexico and Southern Colorado: A Usage-Based Approach. Ph. D,. Thesis.* University of New Mexico.

[B12] Bürkner P. C. (2017). brms: an R package for Bayesian multilevel models using Stan. *J. Statist. Soft.* 80 1–28.

[B13] Buz E. Tanenhaus M. K. Jaeger T. F. (2016). Dynamically adapted context-specific hyper-articulation: feedback from interlocutors affects speakers’ subsequent pronunciations. *J. Memory Lang.* 89 68–86. 10.1016/j.jml.2015.12.009 27375344 PMC4927008

[B14] Bybee J. (1985). *Morphology: A Study of the Relation Between Meaning and Form.* Amsterdam: John Benjamins.

[B15] Bybee J. (1995). Regular morphology and the lexicon. *Lang. Cogn. Proc.* 10 425–455. 10.1080/01690969508407111

[B16] Bybee J. (2001). *Phonology and Language Use.* Cambridge, UK: Cambridge University Press.

[B17] Bybee J. (2002). Word frequency and context of use in the lexical diffusion of phonetically conditioned sound change. *Lang. Variat. Change* 14 261–290. 10.1017/s0954394502143018

[B18] Bybee J. (2017). Grammatical and lexical factors in sound change: a usage-based approach. *Lang. Variat. Change* 29 273–300. 10.1017/s0954394517000199

[B19] Bybee J. Scheibman J. (1999). The effect of usage on degrees of constituency: the reduction of don’t in English. *Linguistics* 37 575–596.

[B20] Blevins J. (2004). *Evolutionary Phonology: The Emergence of Sound Patterns.* Cambridge: Cambridge University Press.

[B21] Chomsky N. Halle M. (1965). Some controversial questions in phonological theory. *J. Linguist.* 1 97–138.

[B22] Coetzee A. W. Pater J. (2011). “13 the place of variation in phonological theory,” in *The Handbook of Phonological Theory*, 2nd Edn, eds Goldsmith J. Riggle J. Yu A. C. L. (Wiley), 401–434. 10.1002/9781444343069.ch13

[B23] Cohen Priva U. (2017). Informativity and the actuation of lenition. *Language* 93 569–597. 10.1353/lan.2017.0037

[B24] Cohen-Goldberg A. M. (2015). Abstract and lexically specific information in sound patterns: evidence from/r/-sandhi in rhotic and non-rhotic varieties of English. *Lang. Speech* 58 522–548. 10.1177/0023830914567168 27483743

[B25] De Jong K. (1998). Stress-related variation in the articulation of coda alveolar stops: flapping revisited. *J. Phonetics* 26 283–310. 10.1006/jpho.1998.0077

[B26] Drummond R. (2018). Maybe it’s a grime [t]ing: th-stopping among urban British youth. *Lang. Soc.* 47 171–196. 10.1017/s0047404517000999

[B27] Edwards J. Beckman M. E. Munson B. (2004). The interaction between vocabulary size and phonotactic probability effects on children’s production accuracy and fluency in nonword repetition. *J. Speech Lang. Hear. Res.* 47 421–436. 10.1044/1092-4388(2004/034) 15157141

[B28] Feldman N. H. Griffiths T. L. Morgan J. L. (2009). The influence of categories on perception: explaining the perceptual magnet effect as optimal statistical inference. *Psychol. Rev.* 116 752–782. 10.1037/a0017196 19839683 PMC2785510

[B29] Fidelholtz J. L. (1975). Word frequency and vowel reduction in English. *Chicago Ling. Soc.* 11 200–213.

[B30] Gardiner S. Nagy N. (2017). Stable variation vs. language change and the factors that constrain them. *Univ.Pennsyl. Working Papers Ling.* 23:10.

[B31] Gahl S. (2008). Time and thyme are not homophones: the effect of lemma frequency on word durations in spontaneous speech. *Language* 84 474–496.

[B32] Gelman A. Hill J. (2007). *Data Analysis Using Regression and Multilevel/Hierarchical Models.* Cambridge: Cambridge University Press.

[B33] German J. S. Carlson K. Pierrehumbert J. B. (2013). Reassignment of consonant allophones in rapid dialect acquisition. *J. Phonetics* 41 228–248. 10.1016/j.wocn.2013.03.001

[B34] Gershkoff-Stowe L. Smith L. B. (1997). A curvilinear trend in naming errors as a function of early vocabulary growth. *Cogn. Psychol.* 34 37–71. 10.1006/cogp.1997.0664 9325009

[B35] Godfrey J. J. Holliman E. C. McDaniel J. (1992). “SWITCHBOARD: telephone speech corpus for research and development,” in *Proceeding of the IEEE International Conference on Acoustics, Speech, and Signal Processing*, 517–520.

[B36] Goldinger S. D. (1998). Echoes of echoes? An episodic theory of lexical access. *Psychol. Rev.* 105 251–279. 10.1037/0033-295x.105.2.251 9577239

[B37] Harmon Z. Barak L. Shafto P. Edwards J. Feldman N. H. (2021). “Making heads or tails of it: a competition–compensation account of morphological deficits in language impairment,” in *Proceedings of the 43rd Annual Conference of the Cognitive Science Society.*

[B38] Harris J. W. Kaisse E. M. (1999). Palatal vowels, glides and obstruents in Argentinian Spanish. *Phonology* 16 117–190. 10.1017/s0952675799003735

[B39] Heathcote A. Brown S. Mewhort D. J. (2000). The power law repealed: the case for an exponential law of practice. *Psychonomic Bull. Rev.* 7 185–207. 10.3758/bf03212979 10909131

[B40] Herd W. Jongman A. Sereno J. (2010). An acoustic and perceptual analysis of/t/and/d/flaps in American English. *J. Phonetics* 38 504–516. 10.1016/j.wocn.2010.06.003

[B41] Honeybone P. (2016). Are there impossible changes? θ> f but f≯θ. *Papers Histor. Phonol.* 1 316–358.

[B42] Hooper J. B. (1976). “Word frequency in lexical diffusion and the source of morphophonological change,” in *Current Progress in Historical Linguistics*, ed. Christie W. (Amsterdam: North-Holland), 96–105. 10.1057/9780230286610_4

[B43] Janda R. D. (2003). “Phonologization” as the start of dephoneticization–or, on sound change and its aftermath: of extension, generalization, lexicalization, and morphologization,” in *The Handbook of Historical Linguistics*, eds Janda R. D. Joseph B. (Wiley), 401–422. 10.1002/9781405166201.ch9

[B44] Kapatsinski V. (2010b). What is it i am writing? Lexical frequency effects in spelling russian prefixes: uncertainty and competition in an apparently regular system. *Corpus Ling. Linguistic Theory* 6 157–215.

[B45] Kapatsinski V. (2010a). Velar palatalization in russian and artificial grammar: constraints on models of morphophonology. *Laboratory Phonol.* 1 361–393.

[B46] Kapatsinski V. (2018a). *Changing Tools Changing Minds: From Learning Theory To Language Acquisition To Language Change.* Cambridge, MA: MIT Press.

[B47] Kapatsinski V. (2018b). “Words versus rules (storage versus online production/processing) in morphology,” in *Oxford Research Encyclopedia of Linguistics*, ed. Aronoff M. (Oxford: Oxford University Press).

[B48] Kapatsinski V. Easterday S. Bybee J. (2020). Vowel reduction: a usage-based perspective. *Rivista di Linguistica* 32 19–44.

[B49] Kleinschmidt D. F. Jaeger T. F. (2015). Robust speech perception: recognize the familiar, generalize to the similar, and adapt to the novel. *Psychol. Rev.* 122 148–203. 10.1037/a0038695 25844873 PMC4744792

[B50] Kraljic T. Samuel A. G. Brennan S. E. (2008). First impressions and last resorts: how listeners adjust to speaker variability. *Psychol. Sci.* 19 332–338. 10.1111/j.1467-9280.2008.02090.x 18399885

[B51] Labov W. (1981). Resolving the neogrammarian controversy. *Language* 57 267–308. 10.2307/413692

[B52] Labov W. (1989). The child as linguistic historian. *Lang. Variat. Change* 1 85–97. 10.1017/s0954394500000120

[B53] Levelt W. J. (1989). *Speaking: From Intention to Articulation.* Cambridge, MA: MIT Press.

[B54] Levelt W. J. Roelofs A. Meyer A. S. (1999). A theory of lexical access in speech production. *Behav. Brain Sci.* 22 1–38. 11301520 10.1017/s0140525x99001776

[B55] Lieberman E. Michel J. B. Jackson J. Tang T. Nowak M. A. (2007). Quantifying the evolutionary dynamics of language. *Nature* 449 713–716. 10.1038/nature06137 17928859 PMC2460562

[B56] Marslen-Wilson W. Nix A. Gaskell G. (1995). Phonological variation in lexical access: abstractness, inference and English place assimilation. *Lang. Cogn. Proc.* 10 285–308. 10.1080/01690969508407097

[B57] Martin A. T. (2007). *The Evolving Lexicon PH. D, Thesis.* UCLA.

[B58] Maye J. Aslin R. N. Tanenhaus M. K. (2008). The weckud wetch of the wast: lexical adaptation to a novel accent. *Cogn. Sci.* 32 543–562. 10.1080/03640210802035357 21635345

[B59] McQueen J. M. Cutler A. Norris D. (2006). Phonological abstraction in the mental lexicon. *Cogn. Sci.* 30 1113–1126. 10.1207/s15516709cog0000_79 21702849

[B60] Motley M. T. Camden C. T. Baars B. J. (1982). Covert formulation and editing of anomalies in speech production: evidence from experimentally elicited slips of the tongue. *J. Verbal Learn. Verbal Behav.* 21 578–594. 10.1016/s0022-5371(82)90791-5

[B61] Mowrey R. Pagliuca W. (1987). “Articulatory evolution,” in *Papers From the 7th International Conference on Historical Linguistics*, eds Ramat A. G. Carruba O. Bernini G. (Amsterdam: John Benjamins), 459–472. 10.1075/cilt.48.34pag

[B62] Mowrey R. Pagliuca W. (1995). The reductive character of articulatory evolution. *Rivista di Linguistica* 7 37–124.

[B63] Navarro D. J. Perfors A. Vong W. K. (2013). Learning time-varying categories. *Memory Cogn.* 41 917–927. 10.3758/s13421-013-0309-6 23606040

[B64] Nielsen K. (2011). Specificity and abstractness of VOT imitation. *J. Phonetics* 39 132–142. 10.1016/j.wocn.2010.12.007

[B65] O’Donnell T. J. (2015). *Productivity and Reuse in Language: A Theory of Linguistic Computation and Storage.* Cambridge, MA: MIT Press.

[B66] Oláh K. Király I. (2019). Young children selectively imitate models conforming to social norms. *Front. Psychol.* 10:1399. 10.3389/fpsyg.2019.01399 PMC6606772 31293474

[B67] Oldfield R. C. Wingfield A. (1965). Response latencies in naming objects. *Quart. J. Exp. Psychol.* 17 273–281. 10.1080/17470216508416445 5852918

[B68] Osthoff H. Brugmann K. (1878). *Morphologische Untersuchungen Auf Den Gebiete Der Indogermanischen Sprachen.* Hirzel S., Germany.

[B69] Peperkamp S. Dupoux E. (2007). “Learning the mapping from surface to underlying representations in an artificial language,” in *Laboratory Phonology 9*, eds Cole J. Hualde J. (Berlin: Mouton de Gruyter), 315–338.

[B70] Perfors A. Tenenbaum J. B. Regier T. (2011). The learnability of abstract syntactic principles. *Cognition* 118 306–338. 10.1016/j.cognition.2010.11.001 21186021

[B71] Phillips B. S. (1984). Word frequency and the actuation of sound change. *Language* 60 320–342. 10.2307/413643

[B72] Phillips B. S. (2001). “Lexical diffusion, lexical frequency, and lexical analysis,” in *Frequency and the Emergence of Linguistic Structure*, eds Bybee J. L. Hopper P. J. (Amsterdam: John Benjamins), 123–136. 10.1075/tsl.45.07phi

[B73] Pierrehumbert J. Granell R. (2018). “On hapax legomena and morphological productivity,” in *Proceedings of the Fifteenth Workshop on Computational Research in Phonetics, Phonology, and Morphology*, 125–130.

[B74] Pierrehumbert J. B. (2001). “Exemplar dynamics: word frequency, lenition and contrast,” in *Frequency and the Emergence of Linguistic Structure*, eds Bybee J. L. Hopper P. J. (Amsterdam: John Benjamins), 137–158. 10.1075/tsl.45.08pie

[B75] Pierrehumbert J. B. (2002). “Word-specific phonetics,” in *Laboratory Phonology 7*, eds Gussenhoven C. Warner N. (Berlin: Mouton de Gruyter), 101–139. 10.1515/9783110197105.1.101

[B76] Raymond W. D. Brown E. L. (2012). “Are effects of word frequency effects of context of use? An analysis of initial fricative reduction in Spanish,” in *Frequency Effects in Language Learning and Processing*, eds Gries S. T. Divjak D. (Berlin: Mouton de Gruyter), 35–52.

[B77] R Core Team (2020). *R: A Language and Environment for Statistical Computing. Software.* R Foundation for Statistical Computing. Available online at: https:// www.R-project.org/

[B78] Schuchardt H. (1885). *Ueber Die Lautgesetze: Gegen Die Junggrammatiker.* Berlin: R. Oppenheim.

[B79] Shport I. A. Johnson G. Herd W. (2018). “Flapping before a stressed vowel – whatever!” in *Proceedings of the Meetings in Acoustics, 31, Paper 060004.*

[B80] Stevens K. N. (1989). On the quantal nature of speech. *J. Phonetics* 17 3–45. 10.1016/s0095-4470(19)31520-7

[B81] Todd S. Pierrehumbert J. B. Hay J. (2019). Word frequency effects in sound change as a consequence of perceptual asymmetries: an exemplar-based model. *Cognition* 185 1–20. 10.1016/j.cognition.2019.01.004 30641466

[B82] Tomaschek F. Tucker B. V. Fasiolo M. Baayen R. H. (2018). Practice makes perfect: The consequences of lexical proficiency for articulation. *Linguist. Vanguard* 4.

[B83] Vaughn C. Kendall T. (2019). Stylistically coherent variants: cognitive representation of social meaning. *Revista de Estudos da Linguagem* 27 1787–1830. 10.17851/2237-2083.0.0.1787-1830

[B84] Warner N. Tucker B. V. (2011). Phonetic variability of stops and flaps in spontaneous and careful speech. *J. Acoust. Soc. Am.* 130 1606–1617. 10.1121/1.3621306 21895098

[B85] Weide B. (1995). *The CMU Pronouncing Dictionary. Version 0.7.*

[B86] Weinreich U. Labov W. Herzog M. I. (1968). “Empirical foundations for a theory of language change,” in *Directions for Historical Linguistics: A Symposium*, ed. Lehmann W. P. (Austin: University of Texas Press), 95–195.

[B87] Wolf M. (2011). “Exceptionality,” in *The Blackwell Companion to Phonology: Phonological Interfaces*, Vol. 4 eds van Oosetendorp M. Ewen C. J. Hume E. Rice K. (Hoboken, NJ: Wiley-Blackwell), 106.

[B88] Xu F. Tenenbaum J. B. (2007). Word learning as Bayesian inference. *Psychol. Rev.* 114 245–272. 17500627 10.1037/0033-295X.114.2.245

[B89] Zeldes A. (2012). *Productivity in Argument Selection.* Berlin: Mouton De Gruyter.

[B90] Zipf G. K. (1935). *The Psycho-Biology of Language.* Oxford: Mifflin Houghton.

[B91] Zuraw K. (2016). Polarized variation. *Catalan J. Ling.* 15 145–171. 10.5565/rev/catjl.185

